# The Chinese EPOCH Measure of Adolescent Wellbeing: Further Testing of the Psychometrics of the Measure

**DOI:** 10.3389/fpsyg.2019.01457

**Published:** 2019-07-03

**Authors:** Guang Zeng, Margaret L. Kern

**Affiliations:** ^1^Department of Psychology, Tsinghua University, Beijing, China; ^2^Melbourne Graduate School of Education, The University of Melbourne, Melbourne, VIC, Australia

**Keywords:** student wellbeing, wellbeing assessment, psychometric analysis, measure development, adolescents

## Abstract

Given the enormous population of Chinese-speaking people worldwide, it is important to establish measures of adolescent wellbeing with adequate evidence for reliability and validity. The EPOCH Measure of Adolescent Wellbeing assesses five positive psychological characteristics (engagement, perseverance, optimism, connectedness, and happiness). An initial study with the English version of the measure found support for a five-factor structure, and evidence for internal reliability, convergence with other wellbeing measures, and divergence across factors and with unrelated constructs. An initial study translated the measure into Chinese and found support for the factor structure of the measure. To further test the measure’s psychometric properties, data were collected from 11 Chinese student samples (*N* = 17,854) from several regions of China. All students completed the EPOCH measure, along with a variety of other measures. In cases where measures overlapped, samples were combined, with relevant sub-sets used to examine convergent and divergent patterns. Confirmatory analyses supported the five-factor structure and factors were internally reliable, but consistency over time was low. The five factors were more strongly correlated with other wellbeing factors than with illbeing factors. While some correlations demonstrated expected convergent and divergent patterns with other constructs, there were also considerable deviations from expected patterns. Norm values specific to the Chinese version of the measure are provided. The study supports the EPOCH measure as a useful cross-sectional tool for measuring adolescent positive functioning, but additional consideration of cross time stability, change, and correlations with other constructs is needed.

## Introduction

The past decade has brought growing interest in and prioritization of youth wellbeing. Assessment of such efforts plays an important role in ensuring that such initiatives add value, providing not only activity, but also impact (White and Kern, 2018). To this end, various measures have been developed. The EPOCH Measure of Adolescent Wellbeing (Kern et al., 2016) is one such measure, capturing five positive psychological characteristics: engagement, perseverance, optimism, connectedness, happiness. A series of 10 studies with Australian and American respondents provided initial psychometric support for the measure. An initial study testing the Chinese version of the measure supported the factor structure of the measure, and also suggested that mean values are not directly comparable across cultures (Kern et al., 2018). The psychometric properties of the measure specifically within Chinese samples need to be further established. Addressing this need, the current study examines evidence for reliability and validity across 11 samples of Chinese adolescents and provides norm values specific to Chinese respondents.

### Adolescent Positive Psychological Functioning

Most parents and teachers, when asked, will say they want their children to be happy (Seligman et al., 2009). Happiness might refer to having a sense of satisfaction, feelings of belonging at school, performing well at school, developing good social character, or a range of other positive aspects. It also means a lack of mental disorder, loneliness, disengagement, and other negative aspects. And yet around the world, mental illness causes considerable disability for young people, and suicide and accidents are the greatest cause of adolescent mortality (World Health Organization, 2017). Twenty to thirty percent of students in the United States are disengaged with learning (Gallup, 2014). Unfortunately, “many of the brightest and most innovative youth will not make it to adulthood or will enter adulthood crippled by chronic disorder” (White and Kern, 2018, p. 3). High rates of mental illness worldwide have spurred concern to find better ways to support young people – helping them to not only manage dysfunction, but also proactively develop positive psychological characteristics that support optimal youth development (Kern et al., 2017). A growing number of curricula and policies have thus been developed worldwide that prioritize youth wellbeing (International Positive Education Network [IPEN], 2017; Kern et al., 2017; Slemp et al., 2017).

Numerous definitions and models of wellbeing exist for both children and adults (Forgeard et al., 2011; Organisation for Economic Co-Operation and Development, 2013; Rose et al., 2017). For our purposes here, we focus on subjective aspects of functioning. Different wellbeing models include a variety of functional aspects. Adolescence is an important period where character, mindsets, attitudes, and skills are being developed that impact subsequent development and functioning. Thus, we focus here not on feelings of happiness, but rather on positive characteristics that support healthy psychosocial development.

Kern et al. (2016) suggested five positive psychological characteristics that support positive youth development: engagement, perseverance, optimism, connectedness, and happiness. Engagement is conceptualized as the capacity to become absorbed in and focused on what one is doing. Perseverance reflects working hard and the ability to keep striving toward one’s goals, despite encountering obstacles. Optimism reflects being hopeful and confident toward the future. Connectedness refers to having the ability to establish satisfying relationships with others, and believing that one is cared for, loved, esteemed, and valued. Happiness reflects having a generally positive mood and feeling content with one’s life.

### The EPOCH Measure

To assess this theoretical model, Kern et al. (2016) developed a 20-item measure, with four items per domain. Through a series of 10 studies with over 4,000 adolescents from the United States and Australia, the authors tested and provided evidence for adequate psychometric properties of the measure. Analyses supported the five-factor structure and internal reliability of the factors, with Engagement being the least reliable factor and Happiness being the most reliable factor. Factors were relatively stable across a 3-week period, with cross-time correlations ranging from *r* = 0.55 to 0.71, with weaker correlations across more distal time points, especially in schools where intervention occurred. Convergent and divergent patterns indicated that the factors were unrelated to age and gender, strongly related to general wellbeing measures, moderately correlated with illbeing measures (e.g., symptoms of depression and anxiety), and differentially correlated with other positive constructs, depending on the factor.

As the measure was developed and tested across two Western cultures, an important question is the extent to which it is applicable to Eastern cultures. To this end, Kern et al. (2018) translated the measure into Chinese and tested the extent to which the measure was invariant across cultures. Comparing a sample of over 3,500 Chinese students with the Australian and US students included in the initial study, models demonstrated weak invariance, indicating support for the five-factor model across cultures, but lacked strong invariance, indicating that mean scores are not directly comparable across cultures. Thus, norm values specific to Chinese respondents are needed. In addition, as the study focused on comparing the factor structure across studies, it did not test internal and cross time reliability nor other aspects of validity, so further testing of the psychometric properties of the measure are needed.

### The Current Study

Given the enormous population of Chinese-speaking people worldwide, it is important to establish measures of adolescent wellbeing with evidence for reliability and validity specifically within Chinese respondents. Replicating the initial study with the Chinese EPOCH measure, we first confirm the EPOCH factor model across 11 new Chinese samples, testing the expected five-factor model. We then test internal and cross-time reliability and consider associations with a variety of other constructs. Finally, we summarize descriptive information for the factors, which provides norm values for the measure.

To consider convergence and divergence with other constructs, we include a variety of measures. Combining the pattern of correlations evident in the original Kern et al. (2016) study and knowledge of characteristics of the Chinese culture, Table 1 summarizes our hypothesized pattern of correlations across the constructs available. First, as the EPOCH factors are positively correlated with one another, we expected a halo effect to occur, such that a positive or negative correlation with one factor would be reflected across the other factors, although with varying strength. Second, as Kern et al.’s (2018) analysis suggested that the Optimism and Happiness factors are more closely aligned with a general wellbeing factor than the other factors, we expected patterns to be similar for Happiness and Optimism, with greater divergence across the Engagement, Perseverance, and Connectedness factors. Relatedly, we expected that Optimism and Happiness would have the strongest associations with other wellbeing factors, with greater variability across the other factors.

**TABLE 1:** Hypothesized range of associations between the five EPOCH factors and other variables measured in one or more samples.

	**E**	**P**	**O**	**C**	**H**
**Demographics**					
Age	−*0.05*, *0.05*	−*0.05*, *0.05*	−*0.05*, *0.05*	−*0.05*, *0.05*	−*0.05*, *0.05*
Gender	−*0.05*, *0.05*	−*0.05*, *0.05*	−*0.05*, *0.05*	*0.00*, *0.10*	−*0.05*, *0.05*
Grade	−*0.05*, *0.05*	−*0.05*, *0.05*	−*0.05*, *0.05*	−*0.05*, *0.05*	−*0.10*, *0.00*
**Other wellbeing-related constructs**					
Coping	0.05, 0.15	0.45, 0.55	0.25, 0.35	0.05, 0.15	0.25, 0.35
Empathy	−0.05, 0.05	−0.05, 0.05	0.05, 0.15	0.50, 0.60	0.05, 0.15
Grit	*0.15*, *0.25*	*0.75*, *0.85*	*0.25*, *0.35*	*0.25*, *0.35*	*0.20*, *0.30*
Growth mindset	*0.05*, *0.15*	*0.25*, *0.35*	*0.20*, *0.30*	*0.20*, *0.30*	*0.15*, *0.25*
Internal regulation	0.40, 0.50	0.50, 0.60	0.05, 0.15	0.05, 0.15	0.25, 0.35
Mastery motivation	0.40, 0.50	0.60, 0.70	0.25, 0.35	0.05, 0.15	0.40, 0.50
Performance motivation	0.40, 0.50	0.60, 0.70	0.25, 0.35	0.25, 0.35	0.05, 0.15
Resilience	0.25, 0.35	0.60, 0.70	0.60, 0.70	0.25, 0.35	0.25, 0.35
School engagement	0.35, 0.45	0.55, 0.65	0.45, 0.55	0.30, 0.40	0.40, 0.50
Self-awareness	0.25, 0.35	0.25, 0.35	0.05, 0.15	0.25, 0.35	0.25, 0.35
Self-efficacy	0.05, 0.15	0.25, 0.35	0.05, 0.15	0.05, 0.15	0.25, 0.35
Work value	0.45, 0.55	0.50, 0.60	0.05, 0.15	0.05, 0.15	0.05, 0.15
**Psychological symptoms**					
Depression	−*0.10*, −*0.20*	−*0.25*, −*0.35*	−*0.35*, −*0.45*	−*0.35*, −*0.45*	−*0.45*, −*0.55*
Anxiety	−*0.00*, *0.05*	−*0.15*, −*0.25*	−*0.25*, −*0.35*	−*0.20*, −*0.30*	−*0.30*, −*0.40*
**Other constructs**					
Physical health	*0.30*, *0.40*	*0.45*, *0.55*	*0.45*, *0.55*	*0.35*, *0.45*	*0.55*, *0.65*
Teacher, parent, peer relationships	*0.20*, *0.30*	*0.35*, *0.45*	*0.45*, *0.55*	*0.50*, *0.60*	*0.45*, *0.55*
School performance	*0.25*, *0.35*	*0.50*, *0.60*	*0.35*, *0.45*	*0.25*, *0.35*	*0.30*, *0.40*

*Predicted range of effect sizes. Italicized ranges are based upon Kern et al. (2016). E = Engagement, P = Perseverance, O = Optimism, C = Connectedness, H = Happiness. See “Materials and Methods” and Supplementary Appendix 1 for details on constructs and scales used.*

Third, we expected to replicate the pattern of results in the original study (Kern et al., 2016). Specifically, the authors found that the factors did not vary across age (*r* < 0.05). Females were slightly more likely to report higher levels of Connectedness (*r* = 0.09), and year level was weakly related with less Happiness (*r* = −0.06). Factors were more strongly correlated with other wellbeing factors than with illbeing factors. We thus expected minimal correlations with age and gender (with weak correlations of gender with Connectedness and Happiness with age), weak-to-moderate negative associations with indicators of psychological distress, and moderate-to-strong positive associations with indicators of wellbeing.

Fourth, we expected Connectedness to be most strongly correlated with relationship variables (teacher, parent, peer, classroom relationships), as theoretically, the ability to connect well with others fosters good relationships with others, and to also correlate with positive social skills (e.g., empathy). We expected that Perseverance, which studies find to be a strong predictor of academic achievement (Duckworth et al., 2007) and positive life, health, and career outcomes (Kern, 2017), would be most strongly correlated with school performance, work value, internalized self-regulation, grit, approach style toward learning, self-regulation, resilience, and coping. Finally, we expected Engagement to be most strongly correlated with school engagement.

## Materials and Methods

### Procedure

Data were collected between August 2016 and April 2018. A survey link was sent to the school administrations of schools that had agreed to be a part of the study. The teachers of each participating class brought their students to their school computer rooms to complete the online survey. Before administering the survey, written consent was obtained from the school administration, teachers, parents, and students at the school. Participants were informed about the objectives of the study and assured that all responses would be kept confidential, only accessible to the research group and used for research purposes. All procedures were approved by the Human Research Ethics Committee at Tsinghua University.

### Participants and Measures

Data came from 11 samples of adolescents from schools across several regions of China including Sichuan Province (Southwestern China), Tianjin City (Northern China), Hunan Province (Southern China), and Shanxi Province (Central China), resulting in a total sample of 17,854 individuals (9,548 male and 8,306 female).

The current analyses used existing data, which add several limits to what we could do in our analyses. To protect student privacy, students were not asked which school they attend. As most samples included two or more schools, we defined samples based on the set of scales completed, rather than based on the school that students attended. As schools managed the consent and administration process, it is unknown how many students within classes might have refused to participate. In addition, the system that was used to collect data only recorded complete responses, such that it is unknown how many students might have started the questionnaire and did not complete it.

All samples completed the 20-item Chinese version of the EPOCH Measure of Adolescent Wellbeing (Kern et al., 2018). Four items assessed each of the five domains on a 1–5 scale (1 = not at all like me, 5 = completely like me). To reduce participant burden while testing a broad array of correlates, students also completed several different scales, as summarized in Table 2 and detailed in Supplementary Appendix 1. In addition, several schools volunteered for their students to complete measures a second time, resulting in 5,459 students (2,827 male and 2,632 female) completing the EPOCH items again, between 3 and 16 months later.

**TABLE 2:** Descriptive information and measures completed for the 11 samples (*N* = 17,854).

	**1**	**2**	**3**	**4**	**5**	**6**	**7**	**8**	**9**	**10**	**11**
**Number of respondents**	778	1,737	1,664	2,129	1,340	1,322	2,493	2,271	2,607	1,279	234
Number of males	383	913	891	1,254	820	688	1,339	1,129	1,346	660	126
Number of females	396	824	773	875	520	634	1,154	1,142	1,261	619	108
Number of schools	2	2	1	1	1	2	3	35	35	10	1
**School type**										
Primary school	X	X	X			X	X	X	X	X	X
Secondary school	X	X				X	X	X	X	X	
Vocational/technical				X	X		X				
**Location**										
Sichuan Province	X					X		X	X	X	X
Tianjin City		X									
Hunan Province			X		X		X				
Shanxi Province				X							
**Area types**										
Urban area	X	X						X	X	X	X
Rural area			X	X	X	X	X				
**Additional measures**										
Academic performance		X	X	X	X	X	X		X	X	
Achievement goal orientation				X		X	X		X		
Anxiety	X	X	X	X	X	X	X	X	X	X	X
Class relationships		X	X	X		X			X		
Coping							X				
Depression	X	X	X	X	X	X	X	X	X	X	X
Empathy							X				
Grit				X	X	X	X		X		
Growth mindset	X	X	X	X	X	X	X		X	X	
Interpersonal skills			X								
Parent relationships			X	X		X			X		X
Peer relationships			X			X					
Physical health	X	X	X		X		X	X		X	X
Resilience	X	X	X	X	X	X			X	X	
School engagement		X	X	X	X	X	X		X	X	
Self-awareness							X				
Self-efficacy						X			X		
Self-regulation	X	X									
Teacher relationships			X	X		X			X		
Work value									X		

*Samples are defined based on the set of measures completed, and thus include multiple schools. All samples completed the EPOCH items, provided age and gender information, along with a series of additional measures, as indicated by X. See Supplementary Appendix 1 for measure details.*

### Data Analyses

In cases where measures overlapped, we combined samples and summarized results across participants, with relevant sub-sets included to examine convergent and divergent patterns. As data came from 11 diverse samples, this provides an integrative data analytic approach in which multiple samples are combined at the item level and analyzed together (Curran and Hussong, 2009; Kern et al., 2014a). As the lack of school identification meant that we were unable to directly test students nested within schools, we conducted analyses for the full sample (ignoring potential nesting effects), and across gender, school type (primary only, primary and secondary, vocational/technical), location (Sichuan Province, Tianjin City, Hunan Province, Shanxi Province), and area (urban or rural). While these analyses do not directly account for the nested structure of the data, it helps to clarify potential boundary conditions of our analyses. Analyses were conducted in R (version 3.4.2).

First, using confirmatory factor analysis (CFA), we evaluated the expected five-factor structure, using the lavaan (Rosseel, 2012) package. Replicating Kern et al. (2018), we used the Satorra–Bentler scaled test statistic (Satorra and Bentler, 1994) and the robust diagonally weighted least squares (DWLS), which uses the polychoric correlation matrix to estimate model parameters to account for the ordinal nature of items. We report the robust maximum-likelihood (ML) and robust DWLS estimates. We evaluated model fit with the Tucker–Lewis Index (TLI), comparative fit index (CFI), root mean square error of approximation (RMSEA) with its 95% confidence interval (CI), and the standardized root mean square residual, with adequate fit indicated by a RMSEA ≤ 0.06 combined with a SRMR ≤ 0.09 and a minimum TLI and CFI of 0.90 (Hu and Bentler, 1999). To test invariance for the sub-groups, we used the semTools package (Jorgensen et al., 2016), which compares increasingly constrained models to the configural model, and focused on change in CFI (ΔCFI). Following Cheung and Rensvold (2002), we considered ΔCFI < 0.01 as indication that the invariance assumption holds.

Second, we tested internal and cross-time consistency. Using the psych package (Revelle, 2016), we estimated four indicators of internal consistency: Cronbach’s α, Guttman’s λ_6_, and minimum and maximum split half reliabilities (β and λ_4_). Cross-time consistency was indicated by Pearson *r* correlation coefficients, tested on the subset of participants that completed the EPOCH items twice.

We then compared mean level differences across gender, school type, location, and area, using independent sample *t*-tests and ANOVA. Finally, we considered convergent and divergent patterns with other constructs, using Pearson *r* correlations for the combined subsets of participants that completed the various measures. With the very large sample size, we focus on the size of the effects rather than statistical significance, considering the extent to which effect size strengths aligned with our hypothesized pattern of results (Table 1).

## Results

Confirmatory analyses generally supported the five-factor structure. Robust ML estimation indicated good fit [^2^(160) = 8,934, CFI = 0.931, TLI = 0.919, RMSEA = 0.055 (CI = 0.055, 0.056), SRMR = 0.041]. Robust DWLS estimation suggested good fit according to RMSEA and SRMR indicators, with weaker fit according to CFI and TLI fit indices [^2^(160) = 10.273, CFI = 0.898, TLI = 0.879, RMSEA = 0.059 (CI = 0.059, 0.060), SRMR = 0.037]. Strict invariance was supported for gender and area, and strong invariance was supported for school type and location (see Supplementary Appendix 2), indicating that the five-factor model is sufficient and mean values are directly comparable across sub-groups.

Factors were internally reliable, but consistency over time was low, ranging from *r* = 0.12 to 0.21 (Table 3). As summarized in Supplementary Appendix 3, males reported higher levels of Engagement [*t*(17,852) = 3.21, *p* < 0.0001] and lower levels of Connectedness [*t*(17,852) = −9.71, *p* < 0.0001] than girls, with no significant differences for the other factors. Respondents from urban areas reported higher levels of all five factors than respondents from rural areas. Respondents from the Sichuan Province or Tianjin City reported higher levels of wellbeing than respondents from the Hunan and Shanxi Provinces. Samples that included both primary and secondary schools reported a higher level of each factor than samples that only included primary schools or vocational and technical schools.

**TABLE 3:** Internal and cross-time reliability.

	**E**	**P**	**O**	**C**	**H**	**Overall**
**Internal reliability** *(Time 1 data, *N* = 17,854)*						
α	0.79	0.80	0.79	0.78	0.89	0.94
λ_6_	0.75	0.75	0.75	0.73	0.86	0.94
λ_4_	0.82	0.81	0.81	0.80	0.90	0.96
β	0.74	0.78	0.77	0.76	0.88	0.86

	**E**	**P**	**O**	**C**	**H**	**Overall**

**Cross-sectional correlations** *(Time 1 above diagonal, Time 2 below diagonal)*						
E	–	0.633	0.597	0.566	0.552	0.802
P	0.605	–	0.628	0.572	0.599	0.821
O	0.608	0.625	–	0.685	0.731	0.871
C	0.562	0.560	0.693	–	0.674	0.835
H	0.549	0.602	0.742	0.678	–	0.853
Overall	0.798	0.810	0.878	0.832	0.858	–

	**E_T2_**	**P_T2_**	**O_T2_**	**C_T2_**	**H_T2_**	**Overall_T2_**

**Cross-time correlations** *(Time 1–Time 2, *N* = 5,429)*						
E_T1_	0.118	0.107	0.084	0.080	0.090	0.115
P_T1_	0.104	0.214	0.110	0.105	0.128	0.158
O_T1_	0.100	0.133	0.178	0.138	0.171	0.172
C_T1_	0.088	0.121	0.137	0.172	0.148	0.159
H_T1_	0.073	0.143	0.153	0.143	0.213	0.174
Overall_T1_	0.115	0.171	0.158	0.153	0.180	0.186

*N = 17,854 at Time 1, 5,429 at Time 2. α, Cronbach’s α; λ_6_, Guttman’s λ_6_; λ_4_, minimum split half; β, maximum split half. Minimum and maximum split halves are based on 10,000 random draws across the data, estimated with the psych package (Revelle, 2016) in R.*

We then examined associations with other constructs (Table 4). As expected, minimal associations occurred between the EPOCH factors and age and gender. The five factors were more strongly correlated with other wellbeing factors than with illbeing factors. Notably, correlations were weaker than we expected for anxiety and depression, but stronger than expected for many of the wellbeing constructs.

**TABLE 4:** Correlations with other constructs.

	***n***	**E**	**P**	**O**	**C**	**H**
**Demographics**						
Age	13, 163	blue−0.018	blue−0.003	blue−0.013	blue−0.006	blue0.002
Gender	17, 854	blue−0.026	blue−0.002	blue0.004	blue0.056	blue0.006
Grade	12, 641	blue0.014	green0.073	green0.067	blue0.000	pink0.067
**Other wellbeing-related constructs**						
Coping	4, 321	green0.49	blue0.543	green0.546	green0.494	green0.557
Empathy	4, 321	green0.427	green0.415	green0.434	pink0.426	green0.414
Grit	13, 427	green0.313	pink0.549	green0.412	blue0.354	green0.41
Growth mindset	7, 757	green0.187	pink0.243	green0.186	pink0.165	blue0.159
*Internal–external motivation*						
Integrated	2, 540	pink0.349	blue0.507	green0.384	green0.354	green0.414
Identified	2, 540	grey0.345	grey0.498	grey0.393	grey0.355	grey0.372
Introjected	2, 540	grey0.188	grey0.224	grey0.195	grey0.195	grey0.155
External	2, 540	grey−0.001	grey−0.019	grey−0.036	grey−0.037	grey−0.061
Mastery motivation						
Approach	10, 325	blue0.424	pink0.553	green0.497	green0.453	blue0.445
Avoidance	10, 325	grey0.208	grey0.213	grey0.196	grey0.179	grey0.139
Performance motivation						
Approach	10, 325	blue0.414	pink0.489	green0.471	green0.415	green0.391
Avoidance	10, 325	grey0.296	grey0.332	grey0.337	grey0.298	grey0.264
Resilience	13, 746	blue0.261	pink0.324	pink0.394	blue0.342	green0.400
School engagement	17, 301	pink0.29	pink0.396	pink0.392	blue0.34	blue0.418
Self-control	1, 716	grey0.374	grey0.433	grey0.399	grey0.368	grey0.347
Self-awareness	4, 321	green0.514	green0.568	green0.601	green0.526	green0.560
Self-efficacy	8, 164	green0.520	green0.590	green0.591	green0.508	green0.521
Work value	8, 162	pink0.330	pink0.467	green0.409	green0.378	green0.386
**Psychological symptoms**						
Depression	17, 854	pink−0.029	pink−0.115	pink−0.166	pink−0.168	pink−0.249
Anxiety	17, 854	blue−0.007	pink−0.088	pink−0.119	pink−0.118	pink−0.199
**Physical health**	9, 662	green0.449	blue0.488	blue0.533	green0.47	pink0.543
**Relationships**						
Teachers	11, 736	blue0.203	pink0.261	pink0.257	pink0.277	pink0.262
Peers	11, 739	pink0.132	pink0.194	pink0.197	pink0.221	pink0.228
Parents	11, 758	pink0.132	pink0.208	pink0.206	pink0.220	pink0.252
Sense of belonging	1, 716	grey0.309	grey0.313	grey0.408	grey0.44	grey0.414
Class relationships	5, 576	grey0.220	grey0.321	grey0.382	grey0.415	grey0.425
Classroom belonging	4, 336	grey0.287	grey0.345	grey0.420	grey0.440	grey0.45
Interpersonal skills	1, 716	grey0.448	grey0.478	grey0.499	grey0.513	grey0.464
**School performance**	15, 120	blue0.346	pink0.432	blue0.388	green0.358	blue0.363

*Number (*n*) varies based on data available from the 11 samples. Blue indicates that the correlation lies within our hypothesized range (Table 1), orange indicates correlations that are weaker than we expected, green indicates correlations that are stronger than we expected, and gray indicates variables that we did not make predictions about. E = Engagement, P = Perseverance, O = Optimism, C = Connectedness, H = Happiness. See Supplementary Appendix 1 for details on constructs and scales used.*

Engagement demonstrated the weakest correlations with other well-being constructs. Correlations matched our expectations for motivation, resilience, anxiety, teacher relationships, and school performance, and were much stronger than expected for coping, empathy, grit, mindset, self-awareness, and self-efficacy. Surprisingly, although Engagement was moderately correlated with school engagement (*r* = 0.29), this correlation was weaker than the correlations between school engagement and the other EPOCH factors.

Consistent with prior studies (e.g., Duckworth et al., 2007; Kern, 2017), Perseverance was strongly correlated with school performance, work value, internalized self-regulation, grit, an approach style toward learning, self-regulation, resilience, and coping. However, associations tended to be weaker than predicted, with the exception of empathy, self-awareness, and self-efficacy, which demonstrated strong correlations with Perseverance. Notably, Perseverance was more strongly correlated with internal forms of motivation and was unrelated to external motivation. Perseverance was also strongly correlated with school engagement and self-control, and was moderately correlated with coping, interpersonal skills, and physical health.

Optimism demonstrated much stronger correlations than we expected with coping, empathy, grit, internal and approach-oriented forms of motivation, self-awareness, self-efficacy, and work value, whereas correlations with resilience, school engagement, and teachers, classroom, and parent relationships were weaker than we expected. Connectedness demonstrated weaker correlations with teacher, peer, and parent relationships and was strongly correlated with interpersonal skills and class belonging/relationships. This suggests that Connectedness captures school belonging more than other forms of belonging. Happiness was strongly correlated with good relationships with teachers, peers, and parents, suggesting that relationships might be a key contributor to happiness. Happiness was strongly correlated with physical health, implying that health and happiness are mutually supportive. Happiness was also highly correlated with resilience and school engagement.

Finally, Table 5 provides descriptive information for each factor, for the full sample and separated by gender, school type, location, and area. As the five-factor model was adequate, factors had acceptable levels of internal reliability and (with some exceptions) followed expected correlational patterns with related and unrelated constructs. We included a large and diverse set of samples, and these values provide norm values that might be expected within Chinese students.

**TABLE 5:** EPOCH norm values, for the full combined sample, and by gender, school type, location, and area.

	***N***	***M***	***SD***	**Quartiles**				
				**Min**	**25%**	**50%**	**75%**	**Max**
**Engagement**								
Full sample	17,854	3.66	0.89	1.00	3.00	3.75	4.25	5.00
Males	9,548	3.68	0.91	1.00	3.00	3.75	4.50	5.00
Females	8,306	3.64	0.87	1.00	3.00	3.75	4.25	5.00
Primary	1,898	3.33	0.94	1.00	2.50	3.25	4.00	5.00
Primary/secondary	12,487	3.76	0.90	1.00	3.00	3.75	4.50	5.00
Vocation/technical	3,469	3.50	0.73	1.00	3.00	3.50	4.00	5.00
Sichuan Province	8,491	3.76	0.90	1.00	3.00	3.75	4.50	5.00
Tianjin City	1,737	3.66	0.98	1.00	3.00	3.75	4.50	5.00
Hunan Province	5,497	3.53	0.88	1.00	2.00	3.50	4.25	5.00
Shanxi Province	2,129	3.60	0.75	1.00	3.00	3.50	4.00	5.00
Urban	8,906	3.78	0.90	1.00	3.25	3.75	4.50	5.00
Rural	8,948	3.54	0.86	1.00	3.00	3.50	4.25	5.00
**Perseverance**								
Full sample	17,854	3.77	0.85	1.00	3.25	3.75	4.50	5.00
Males	9,548	3.78	0.86	1.00	3.25	3.75	4.50	5.00
Females	8,306	3.77	0.84	1.00	3.25	3.75	4.50	5.00
Primary	1,898	3.42	0.92	1.00	2.75	3.50	4.25	5.00
Primary/secondary	12,487	3.87	0.86	1.00	3.25	4.00	4.50	5.00
Vocation/technical	3,469	3.61	0.70	1.00	3.00	3.50	4.00	5.00
Sichuan Province	8,491	3.88	0.86	1.00	3.25	4.00	4.50	5.00
Tianjin City	1,737	3.91	0.89	1.00	3.25	4.00	4.75	5.00
Hunan Province	5,497	3.59	0.83	1.00	3.00	3.50	4.25	5.00
Shanxi Province	2,129	3.70	0.73	1.00	3.25	3.75	4.25	5.00
Urban	8,906	3.91	0.86	1.00	3.25	4.00	4.75	5.00
Rural	8,948	3.64	0.82	1.00	3.00	3.75	4.25	5.00
**Optimism**								
Full sample	17,854	3.97	0.87	1.00	3.50	4.00	4.75	5.00
Males	9,548	3.96	0.88	1.00	3.25	4.00	4.75	5.00
Females	8,306	3.98	0.85	1.00	3.50	4.00	4.75	5.00
Primary	1,898	3.76	0.90	1.00	3.00	3.75	4.50	5.00
Primary/secondary	12,487	4.07	0.87	1.00	3.50	4.25	4.75	5.00
Vocation/technical	3,469	3.71	0.77	1.00	3.25	3.75	4.25	5.00
Sichuan Province	8,491	4.09	0.87	1.00	3.50	4.25	4.75	5.00
Tianjin City	1,737	4.04	0.92	1.00	3.50	4.25	4.75	5.00
Hunan Province	5,497	3.83	0.85	1.00	3.25	3.75	4.50	5.00
Shanxi Province	2,129	3.83	0.80	1.00	3.25	4.00	4.50	5.00
Urban	8,906	4.10	0.86	1.00	3.50	4.25	4.75	5.00
Rural	8,948	3.84	0.85	1.00	3.25	4.00	4.50	5.00
**Connectedness**								
Full sample	17,854	4.05	0.83	1.00	3.50	4.25	4.75	5.00
Males	9,548	4.00	0.86	1.00	3.50	4.00	4.75	5.00
Females	8,306	4.12	0.80	1.00	3.50	4.25	4.75	5.00
Primary	1,898	3.83	0.90	1.25	3.25	4.00	4.50	5.00
Primary/secondary	12,487	4.14	0.83	1.00	3.75	4.25	4.75	5.00
Vocation/technical	3,469	3.85	0.74	1.00	3.25	4.00	4.50	5.00
Sichuan Province	8,491	4.13	0.83	1.00	3.75	4.25	5.00	5.00
Tianjin City	1,737	4.08	0.89	1.00	3.50	4.25	4.75	5.00
Hunan Province	5,497	3.96	0.83	1.00	3.50	4.00	4.75	5.00
Shanxi Province	2,129	3.95	0.75	1.00	3.50	4.00	4.50	5.00
Urban	8,906	4.15	0.84	1.00	3.75	4.25	5.00	5.00
Rural	8,948	3.96	0.82	1.00	3.50	4.00	4.75	5.00
**Happiness**								
Full sample	17,854	4.05	0.91	1.00	3.50	4.25	5.00	5.00
Males	9,548	4.04	0.92	1.00	3.25	4.25	5.00	5.00
Females	8,306	4.06	0.89	1.00	3.50	4.25	5.00	5.00
Primary	1,898	3.93	0.92	1.00	3.25	4.00	4.75	5.00
Primary/secondary	12,487	4.13	0.92	1.00	3.50	4.50	5.00	5.00
Vocation/technical	3,469	3.81	0.80	1.00	3.25	4.00	4.50	5.00
Sichuan Province	8,491	4.14	0.93	1.00	3.50	4.50	5.00	5.00
Tianjin City	1,737	4.15	0.95	1.00	3.75	4.50	5.00	5.00
Hunan Province	5,497	3.91	0.88	1.00	3.25	4.00	4.75	5.00
Shanxi Province	2,129	3.95	0.80	1.00	3.25	4.00	4.75	5.00
Urban	8,906	4.16	0.92	1.00	3.50	4.50	5.00	5.00
Rural	8,948	3.94	0.88	1.00	3.25	4.00	4.75	5.00
**Overall wellbeing**								
Full sample	17,854	3.90	0.73	1.00	3.40	3.95	4.45	5.00
Males	9,548	3.89	0.75	1.00	3.35	3.95	4.50	5.00
Females	8,306	3.91	0.70	1.00	3.45	4.00	4.45	5.00
Primary	1,898	3.65	0.76	1.35	3.09	3.65	4.25	5.00
Primary/secondary	12,487	4.00	0.73	1.00	3.55	4.10	4.55	5.00
Vocation/technical	3,469	3.70	0.61	1.00	3.25	3.65	4.05	5.00
Sichuan Province	8,491	4.00	0.73	1.00	3.55	4.10	4.55	5.00
Tianjin City	1,737	3.97	0.77	1.00	3.50	4.10	4.55	5.00
Hunan Province	5,497	3.76	0.71	1.00	3.25	3.75	4.30	5.00
Shanxi Province	2,129	3.81	0.63	1.00	3.38	3.80	4.25	5.00
Urban	8,906	4.02	0.73	1.00	3.55	4.15	4.60	5.00
Rural	8,948	3.78	0.71	1.00	3.30	3.80	4.30	5.00

## Discussion

The past few decades have brought growing interest globally in individual and societal wellbeing. One-fifth of the world’s population – over 1.4 billion people – live in China (United Nations, 2017), and Chinese (all dialects) is the most spoken language in the world (Noack and Gamio, 2015). It can be questionable to compare self-reported Likert-style questionnaires across cultures (Heine et al., 2002). As such, having valid instruments of wellbeing with sound psychometrics and norms specific to Chinese populations is important.

The current study tested the Chinese version of the EPOCH Measure of Adolescent Well-Being (Kern et al., 2018) across 17,854 Chinese adolescents from 11 samples from urban and rural areas in the Northern, Southern, and Central parts of China. CFAs supported the five-factor structure of the measure, factors were internally consistent, and factors generally demonstrated expected convergent and divergent correlations with similar and dissimilar constructs. Our results further support the EPOCH measure as a useful tool for measuring adolescent positive functioning for Chinese adolescents, but also raise additional questions around the appropriate uses and applications of the measure.

While definitions and models of positive psychological functioning vary, most models include multiple domains and indicators (Forgeard et al., 2011; Friedman and Kern, 2014). Some recent studies have questioned whether wellbeing indeed comprises more than one dimension (e.g., Goodman et al., 2017). Our results add support for multidimensional models of student psychological functioning. Statistically, adequate to good fit for the five-factor model occurred, depending on the estimation approach and fit index. Happiness consistently demonstrated the strongest correlations, which may reflect a halo effect (i.e., when you feel good in one area, you also feel good across a range of other areas), or may be a result of the greater internal consistency of the factor items compared to the other factors.

While some constructs correlated strongly across the EPOCH factors, with little differentiation by domain (e.g., coping, empathy, approach motivation, physical health), other correlates demonstrated more differential patterns. For instance, Perseverance demonstrated stronger correlations than the other factors with grit, self-control, and school performance. Self-efficacy and self-awareness were most strongly correlated with Perseverance and Optimism. Connectedness demonstrated the strongest correlations with several relationship variables. Happiness demonstrated the strongest correlations with resilience and the absence of psychological symptoms. Still, the differences among these correlations were small (generally *r* < 0.10). All correlates were self-reported, which can add method variance and conflate correlational patterns. Alternatively, the measure may be inadequate for detecting specific differential patterns. Future studies might further test the convergent and divergent patterns, using a variety of assessment methods and specific correlates that ought to have divergent associations with each factor.

While the measure may be inadequate for identifying distinctive patterns of each factor, there can still be practical benefits of multidimensional wellbeing models (e.g., Kern et al., 2014b; McQuaid and Kern, 2017). By considering multiple dimensions of psychological functioning, it can identify areas of strength and weaknesses, informing more targeted interventions and programs to best support young people. An analogy can be seen with indicators of academic performance. Although grade point average provides an overall summary score of how a young person is performing, it provides little indication of which subjects a student is exceling or struggling with. The profile of marks across different subjects is more useful. The measure does provide a profile of responses across five domains, adding a greater picture of psychological functioning compared to when relying on a single overall wellbeing score alone.

The pattern of correlations with other constructs suggests that the five factors together provide an indication of students who are interested in learning (valuing work, motivated to learn) and have strong social and emotional skills (aware of themselves, empathetic toward others, self-controlled, coping well).

As expected, the EPOCH domains were minimally correlated with age and gender, and the model was invariant across gender and school type. The EPOCH domains were specifically chosen to be stable across development, such that scores reflect psychological functioning rather than maturation effects (Kern et al., 2016). The minimal correlations with age suggest that the factors are indeed capturing something more than developmental change, supporting the use of the measure across the adolescent years. Future studies might test the extent to which the measure can be used at younger or older ages. It also appears to be appropriate for both boys and girls.

Unexpectedly, although Engagement was correlated with school engagement, the effect sizes were weaker than for the other EPOCH dimensions. Engagement is a multidimensional dimension, defined in some models across cognitive, affective, and behavioral domains (Appleton et al., 2008), and in other models defined as vigor, absorption, and dedication (Upadyaya and Salmela-Aro, 2013). The two engagement measures appear to capture different concepts of engagement. The EPOCH Engagement items focus primarily on the cognitive domain, capturing elements of flow (e.g., absorption in tasks). Indeed, the factor was strongly correlated with internal, approach-oriented forms of motivation, self-awareness, and self-efficacy. In contrast, school engagement was most strongly correlated with Optimism and Perseverance, reflecting vigor and dedication.

Interestingly, Perseverance was strongly correlated with integrated regulation (i.e., the most internalized form of motivation), with weaker correlations for more externalized forms of regulation (identified, introjected, and external). The other factors similarly showed a decreasing correlational pattern from internalized to externalized forms of regulation. Self-determination theory suggests that more intrinsic forms of motivation support the satisfaction of basic needs for relatedness, autonomy, and competence, which in turn supports greater wellbeing (Ryan and Deci, 2000; Ryan and Deci, 2001). It may be that intrinsic forms of motivation help support the development of positive psychological characteristics, or that the EPOCH factors help enable basic needs to be fulfilled, subsequently resulting in greater wellbeing and better performance at school.

Although responses were spread across the distribution for each factor, responses were biased toward the maximum value for the Optimism, Connectedness, and Happiness factors, suggesting potential ceiling effects of the measure. It is possible that respondents were indeed high functioning, or that the five-point scale is insufficient to detect differences in wellbeing domains. The number of scale points that are needed for measures is an area of continued contention. While a limited number of scale points reduces the cognitive complexity of the items, it also might prevent individual differences and changes in positive psychological functioning from being detected. This also might speak to some of the lower cross-time reliabilities, where students at the ceiling might only be able to stay level or decline. Future studies might consider the impact of using a different number of scale points and the implications of the skewed nature of the factors.

While the results support the use of the measure for cross-sectional assessment, it is unclear whether the measure is appropriate for tracking stability and change over time or for testing the impact of interventions. Cross-time consistencies were low, with Happiness being most consistent and Engagement being least consistent. Kern et al. (2016) similarly found mixed evidence for stability, with greater stability apparent over brief periods, and less stability over time. In the current study, the lag between measurement occasions ranged from 3 to 16 months. Several schools also simultaneously introduced a number of positive psychology initiatives and programs, such that some students might be expected to change, and others would not, creating considerable noise in the data. Unfortunately, data were not available on what schools did or did not do between times of assessment. Alternatively, it could be issues with the measure itself. The extent to which low cross-time correlations were due to instability of the measure, actual changes that occurred, whether or not intervention occurred, length of time between assessments, or other confounding variables, is unclear. Future research might further test the extent to which temporal, intervention, and other factors impact the stability of EPOCH scores and determine whether or not the measure is appropriate for evaluating interventions.

### Limitations

We included a diverse set of participants, with 11 different samples across urban and rural places in various parts of China. However, the samples are not nationally representative and were dependent upon the schools that were willing to be involved in the assessment. Although models were consistent across gender, location, and school type, with the diversity of Chinese dialects and sub-cultures, additional consideration of cultural variations across different samples living within and outside of China is needed. While we provide norm values based on the samples included, the extent to which these norms may or may not be relevant to other Chinese sub-cultures is needed. As the current study employed self-report measures, common method variance could be a concern. Future work might combine the self-reported measure with objective physical and academic measures to achieve a more comprehensive perspective of these psychological constructs.

## Conclusion

To move beyond treating psychopathology in a reactive manner, proactively supporting positive youth development is a necessary goal. Measurement plays an important role in understanding and building wellbeing. The current study provides support for the use of the Chinese EPOCH measure for assessing levels of adolescent positive psychological functioning in Chinese adolescents, adding to the toolbox of valid measures, and also points to the need for studies that determine the best measures for repeated measurement and intervention evaluation.

## Ethics Statement

This study was carried out in accordance with the recommendations of Research Ethics Committee in Psychology Department, Tsinghua University, with written informed consent from all subjects. All subjects gave written informed consent in accordance with the Declaration of Helsinki. The protocol was approved by the Tsinghua University Research Ethics Committee. The study used an online questionnaire to investigate positive psychological functioning. Parents and students provided informed consent, and no valuable populations were included. As such, the study and was considered minimal risk study.

## Author Contributions

GZ and MK designed the study and wrote the manuscript. GZ collected the data and MK conducted the analyses.

## Conflict of Interest Statement

The authors declare that the research was conducted in the absence of any commercial or financial relationships that could be construed as a potential conflict of interest.

## References

[B1] Appleton J. J. Christenson S. L. Furlong M. J. (2008). Student engagement with school: Critical conceptual and methodological issues of the construct. *Psychology in the Schools* 45 369–386.

[B2] Cheung G. W. Rensvold R. B. (2002). Evaluating goodness-of-fit indexes for testing measurement invariance. *Structural Equation Modeling* 9 233–255. 10.1097/NNR.0b013e3182544750 22551991 PMC3361901

[B3] Curran P. J. Hussong A. M. (2009). Integrative data analysis: The simultaneous analysis of multiple data sets. *Psychological Methods* 14 81–100. 10.1037/a0015914 19485623 PMC2777640

[B4] Duckworth A. L. Peterson C. Matthews M. D. Kelly D. R. (2007). Grit: Perseverance and passion for long-term goals. *Personality Processes and Individual Differences* 92 1087–1101. 10.1037/0022-3514.92.6.1087 17547490

[B5] Forgeard M. J. C. Jayawickreme E. Kern M. L. Seligman M. E. P. (2011). Doing the right thing: Measuring well-being for public policy. *International Journal of Wellbeing* 1 79–106.

[B6] Friedman H. S. Kern M. L. (2014). Personality, well-being, and health. *Annual Review of Psychology* 65 719–742. 10.1146/annurev-psych-010213-115123 24405364

[B7] Gallup. (2014). *Gallup student poll 2014 U.S. overall report: Measuring non-cognitive metrics that predict student success.* Available at: https://www.gallup.com/services/180029/gallup-student-poll-2014-overall-report.aspx (accessed December 11, 2014).

[B8] Goodman F. Disabato D. Kashdan T. Kauffman S. (2017). Measuring well-being: A comparison of subjective wellbeing and PERMA. *J. Posit. Psychol.* 13 321–332. 10.1080/17439760.2017.1388434

[B9] Heine S. J. Lehman D. R. Peng K. Greenholtz J. (2002). What’s wrong with cross-cultural comparisons of subjective Likert scales?: The reference-group effect. *Journal of Personality and Social Psychology* 82 903–918. 10.1037//0022-3514.82.6.903 12051579

[B10] Hu L. Bentler P. M. (1999). Cutoff criteria for fit indexes in covariance structure analysis: conventional criteria versus new alternatives. *Struct. Equ. Modeling* 6 1–55. 10.1080/10705519909540118

[B11] International Positive Education Network [IPEN]. (2017). *The state of positive education: A review of history, policy, practice, and research.* Availalable at: https://worldgovernmentsummit.org/api/publications/document/8f647dc4-e97c-6578-b2f8-ff0000a7ddb6

[B12] Jorgensen T. D. Pornprasertmanit S. Miller P. Schoemann A. Rosseel Y. (2016). *Useful tools for structural equation modeleing: Package ‘semTools’* (version 0.4*–*14). Available at: https://github.com/simsem/semTools/wiki (accessed September 24, 2018).

[B13] Kern M. L. (2017). *Perseverance, achievement, and positive education. In M. A. White, G. R. Slemp, & A. S. Murray (Eds.), Future directions in well-being: Education, organizations, and policy* (pp. 75-79). Switzerland: Springer Nature.

[B14] Kern M. L. Benson L. Steinberg E. A. Steinberg L. (2016). The EPOCH measure of adolescent well-being. *Psychological Assessment* 28 586–597. 10.1037/pas0000201 26302102

[B15] Kern M. L. Hampson S. E. Goldberg L. R. Friedman H. S. (2014a). Integrating prospective longitudinal data: Modeling personality and health in the Terman Life Cycle and Hawaii Longitudinal studies. *Developmental Psychology* 50 1390–1406. 10.1037/a0030874 23231689 PMC3758911

[B16] Kern M. L. Park N. Peterson C. Romer D. (2017). *The positive perspective on youth development. In D. Romer & the Commission Chairs of the Adolescent Mental Health Initiative of the Annenberg Public Policy Center and the Sunnylands Trust (Eds.), Treating and preventing adolescent mental disorders: What we know and what we don’t know*, Vol. 2 New York, NY: Oxford University Press, 543–567.

[B17] Kern M. L. Waters L. White M. Adler A. (2014b). Assessing employee wellbeing in schools using a multifaceted approach: Associations with physical health, life satisfaction, and professional thriving. *Psychology* 5 500–513. 10.4236/psych.2014.56060

[B18] Kern M. L. Zeng G. Hou H. Peng K. (2018). The Chinese version of the EPOCH Measure of Adolescent Wellbeing: Testing cross-cultural measurement invariance. *J. Psychoeduc. Assess.* 10.1177/0734282918789561

[B19] McQuaid M. Kern P. (2017). *Your wellbeing blueprint: Feeling good and doing well at work.* Victoria, VIC Australia: McQuaid Ltd.

[B20] Noack R. Gamio L. (2015). *The world’s languages, in* 7 maps and charts”. *The Washington Post.* Washington DC Available at: https://wapo.st/1QmH08Y?tid=ss_mail&utm_term=.71df8de1b1d0

[B21] Organisation for Economic Co-Operation and Development (2013). *OECD Guidelines on Measuring Subjective Well-Being.* Paris: OECD Publishing, 265 10.1787/9789264191655-en 24600748

[B22] Revelle W. (2016). *psych: Procedures for Psychological, Psychometric, and Personality Research, Version 1.8.12.* Available at: https://cran.r-project.org/web/packages/psych/index.html

[B23] Rose T. Joe S. Williams A. Harris R. Betz G. Stewart-Brown S. (2017). Measuring mental well-being among adolescents: a systematic review of instruments. *J. Child Fam. Stud.* 26 2349–2362. 10.1007/s10826-017-0754-0

[B24] Rosseel Y. (2012). lavaan: an R package for structural equation modeling. *J. Stat. Softw.* 48 1–36. 10.18637/jss.v048.i02

[B25] Ryan R. M. Deci E. L. (2000). Self-determination theory and the facilitation of intrinsic motivation, social development, and well-being. *American Psychologist* 55 68–78. 11392867 10.1037//0003-066x.55.1.68

[B26] Ryan R. M. Deci E. L. (2001). On happiness and human potentials: a review of research on hedonic and eudaimonic well-being. *Annu. Rev. Psychol.* 52 141–166. 10.1146/annurev.psych.52.1.141 11148302

[B27] Satorra A. Bentler P. M. (1994). “Corrections to test statistics and standard errors in covariance structure analysis,” in *Latent Variables Analysis: Applications for Developmental Research*, eds von Eye A. Clogg C. C. (Thousand Oaks, CA: Sage), 399–419.

[B28] Seligman M. E. P. Ernst R. M. Gillham J. Reivich K. Linkins M. (2009). Positive education: Positive psychology and classroom interventions. *Oxford Review of Education* 35 293–311. 10.1080/03054980902934563

[B29] Slemp G. R. Chin T. C. Kern M. L. Siokou C. Loton D. Oades L. G. (2017). *Positive education in Australia: Practice, measurement, and future directions. In E. Fryenberg, A. J. Martin, & R. J. Collie (Eds.), Social and emotional learning in Australia and the Asia Pacific.* Singapore: Springer, 101–122.

[B30] United Nations. (2017). *World Population Prospects*: *The 2017 Revision*. *UN Department of Economic and Social Affairs, Population Division.* Available at: https://population.un.org/wpp/

[B31] Upadyaya K. Salmela-Aro K. (2013). Development of school engagement in association with academic success and well-being in varying social contexts: A review of empirical research. *European Psychologist* 18 136–147. 10.1027/1016-9040/a000143)

[B32] White M. Kern M. L. (2018). Positive education: Learning and teaching for wellbeing and academic mastery. *Int. J. Wellbeing* 8 10.5502/ijw.v8i1.588

[B33] World Health, and Organization. (2017). *Depression and other common mental disorders: Global health estimates.* Available at: http://www. who.int/mental_health/management/depression/prevalence_global_health_ estimates/en/

