# Supplementary material for: The Chinese EPOCH Measure of Adolescent Wellbeing: Further Testing of the Psychometrics of the Measure
Source: Front Psychol. 2019 Jul 3;10:1457. doi: 10.3389/fpsyg.2019.01457 (PMC6617957; doi:10.3389/fpsyg.2019.01457)
Supplement: Supplementary file 1 [file Data_Sheet_1.doc]

**Appendix 1**

*Source Information for Scales and Measures Used in the 11 Samples*

| **Domain** | **Source** |
| --- | --- |
| **1) Sample 1** | |
| Age | Single item |
| Gender | Single item |
| Grade | Single item |
| School | Single item |
| School Type | Single item |
| Engagement | EPOCH measure (Kern et al., 2016) |
| Perseverance | EPOCH measure (Kern et al., 2016) |
| Optimism | EPOCH measure (Kern et al., 2016) |
| Connectedness | EPOCH measure (Kern et al., 2016) |
| Happiness | EPOCH measure (Kern et al., 2016) |
| External regulation | SRQ – A (Ryan & Connell, 1989) |
| Introjected regulation | SRQ – A (Ryan & Connell, 1989) |
| Identified regulation | SRQ – A (Ryan & Connell, 1989) |
| Integrated regulation | SRQ – A (Ryan & Connell, 1989) |
| Resilience | Brief Resilience Scale (Smith et al., 2008) |
| Growth mindset | Growth mindset inventory (Dweck, 1996) |
| Anxiety | PROMIS pediatric short form (Varni et al., 2014) |
| Depression | PROMIS pediatric short form (Varni et al., 2014) |
| Physical vitality | Healthy Pathways (Bevans et al., 2010) |
| **2) Sample 2** | |
| Age | Single item |
| Gender | Single item |
| Grade | Single item |
| School | Single item |
| School Type | Single item |
| Engagement | EPOCH measure (Kern et al., 2016) |
| Perseverance | EPOCH measure (Kern et al., 2016) |
| Optimism | EPOCH measure (Kern et al., 2016) |
| Connectedness | EPOCH measure (Kern et al., 2016) |
| Happiness | EPOCH measure (Kern et al., 2016) |
| External regulation | SRQ – A (Ryan & Connell, 1989) |
| Introjected regulation | SRQ – A (Ryan & Connell, 1989) |
| Identified regulation | SRQ – A (Ryan & Connell, 1989) |
| Integrated regulation | SRQ – A (Ryan & Connell, 1989) |
| School engagement | Healthy Pathways (Bevans et al., 2010) |
| Grit | Grit scale (Duckworth et al., 2007) |
| Belonging to class | Relatedness Scale (Furrer & Skinner, 2003) |
| School performance | Healthy Pathways (Bevans et al., 2010) |
| Resilience | Brief Resilience Scale (Smith et al., 2008) |
| Growth mindset | Growth mindset inventory-8 item (Dweck, 1996) |
| Anxiety | PROMIS pediatric short form (Varni et al., 2014) |
| Depression | PROMIS pediatric short form (Varni et al., 2014) |
| Health | PROMIS pediatric short form (Varni et al., 2014) |
| **3) Sample 3** | |
| Age | Single item |
| Gender | Single item |
| Grade | Single item |
| School | Single item |
| School Type | Single item |
| Engagement | EPOCH measure (Kern et al., 2016) |
| Perseverance | EPOCH measure (Kern et al., 2016) |
| Optimism | EPOCH measure (Kern et al., 2016) |
| Connectedness | EPOCH measure (Kern et al., 2016) |
| Happiness | EPOCH measure (Kern et al., 2016) |
| School engagement | Healthy Pathways (Bevans et al., 2010) |
| Grit | Grit scale (Duckworth et al., 2007) |
| Relatedness-mother | Sense of relatedness (Furrer et al.,2003) |
| Relatedness-father | Sense of relatedness (Furrer et al.,2003) |
| Relatedness-teacher | Sense of relatedness (Furrer et al.,2003) |
| Relatedness-class | Sense of relatedness (Furrer et al.,2003) |
| Relatedness-friend | Sense of relatedness (Furrer et al.,2003) |
| School performance | Sense of relatedness (Furrer et al.,2003) |
| Resilience | Brief Resilience Scale (Smith et al., 2008) |
| Growth mindset | Growth mindset inventory-8 item (Dweck, 1996) |
| Initiation | 5 domains of interpersonal competence in peer relationships (Buhrmester et al., 1988） |
| Negative Assertion | 5 domains of interpersonal competence in peer relationships (Buhrmester et al., 1988） |
| Exposure | 5 domains of interpersonal competence in peer relationships (Buhrmester et al., 1988） |
| Emotional Support | 5 domains of interpersonal competence in peer relationships (Buhrmester et al., 1988） |
| Self-Control | 5 domains of interpersonal competence in peer relationships (Buhrmester et al., 1988） |
| Interpersonal Skill | 5 domains of interpersonal competence in peer relationships (Buhrmester et al., 1988） |
| Anxiety | PROMIS pediatric short form (Varni et al., 2014) |
| Depression | PROMIS pediatric short form (Varni et al., 2014) |
| Health | PROMIS pediatric short form (Varni et al., 2014) |
| **4) Sample 4** | |
| Age | Single item |
| Gender | Single item |
| Grade | Single item |
| School | Single item |
| School Type | Single item |
| Engagement | EPOCH measure (Kern et al., 2016) |
| Perseverance | EPOCH measure (Kern et al., 2016) |
| Optimism | EPOCH measure (Kern et al., 2016) |
| Connectedness | EPOCH measure (Kern et al., 2016) |
| Happiness | EPOCH measure (Kern et al., 2016) |
| Grit | Grit scale (Duckworth et al., 2007) |
| Mastery-Approach | 2 × 2 achievement goal framework (Elliot et al., 2001) |
| Mastery-Avoidance | 2 × 2 achievement goal framework (Elliot et al., 2001) |
| Performance-Approach | 2 × 2 achievement goal framework (Elliot et al., 2001) |
| Performance-Avoidance | 2 × 2 achievement goal framework (Elliot et al., 2001) |
| School engagement | Healthy Pathways (Bevans et al., 2010) |
| Academic performance | Healthy Pathways (Bevans et al., 2010) |
| Teacher Relation | Inclusion of others in the self scale (Aron et al., 1992) |
| Classroom Relation | Inclusion of others in the self scale (Aron et al., 1992) |
| Parent Relation | Inclusion of others in the self scale (Aron et al., 1992) |
| Resilience | Brief Resilience Scale (Smith et al., 2008) |
| Growth mindset | Growth mindset inventory (Dweck, 1996) |
| Anxiety | PROMIS pediatric short form (Varni et al., 2014) |
| Depression | PROMIS pediatric short form (Varni et al., 2014) |
| **5) Sample 5** | |
| Age | Single item |
| Gender | Single item |
| Grade | Single item |
| Major | Single item |
| School | Single item |
| School Type | Single item |
| Engagement | EPOCH measure (Kern et al., 2016) |
| Perseverance | EPOCH measure (Kern et al., 2016) |
| Optimism | EPOCH measure (Kern et al., 2016) |
| Connectedness | EPOCH measure (Kern et al., 2016) |
| Happiness | EPOCH measure (Kern et al., 2016) |
| Grit | Grit scale (Duckworth et al., 2007) |
| School engagement | Healthy Pathways (Bevans et al., 2010) |
| Academic performance | Healthy Pathways (Bevans et al., 2010) |
| Resilience | Brief Resilience Scale (Smith et al., 2008) |
| Growth mindset | Growth mindset inventory (Dweck, 1996) |
| Anxiety | PROMIS pediatric short form (Varni et al., 2014) |
| Depression | PROMIS pediatric short form (Varni et al., 2014) |
| Health | PROMIS pediatric short form (Varni et al., 2014) |
| **6) Sample 6** | |
| Age | Single item |
| Gender | Single item |
| Grade | Single item |
| School | Single item |
| School Type | Single item |
| Engagement | EPOCH measure (Kern et al., 2016) |
| Perseverance | EPOCH measure (Kern et al., 2016) |
| Optimism | EPOCH measure (Kern et al., 2016) |
| Connectedness | EPOCH measure (Kern et al., 2016) |
| Happiness | EPOCH measure (Kern et al., 2016) |
| Grit | Grit scale (Duckworth et al., 2007) |
| Mastery-Approach | 2 × 2 achievement goal framework (Elliot et al., 2001) |
| Mastery-Avoidance | 2 × 2 achievement goal framework (Elliot et al., 2001) |
| Performance-Approach | 2 × 2 achievement goal framework (Elliot et al., 2001) |
| Performance-Avoidance | 2 × 2 achievement goal framework (Elliot et al., 2001) |
| School engagement | Healthy Pathways (Bevans et al., 2010) |
| Academic performance | Healthy Pathways (Bevans et al., 2010) |
| Teacher Relation | Inclusion of others in the self scale (Aron et al., 1992) |
| Classroom Relation | Inclusion of others in the self scale (Aron et al., 1992) |
| Parent Relation | Inclusion of others in the self scale (Aron et al., 1992) |
| Belonging to class | Relatedness Scale (Furrer & Skinner, 2003) |
| Self-Efficacy | The GSES (Sherer & Adams, 1983) |
| Resilience | Brief Resilience Scale (Smith et al., 2008) |
| Growth mindset | Growth mindset inventory (Dweck, 1996) |
| Anxiety | PROMIS pediatric short form (Varni et al., 2014) |
| Depression | PROMIS pediatric short form (Varni et al., 2014) |
| **7) Sample 7** | |
| Age | Single item |
| Gender | Single item |
| Grade | Single item |
| School | Single item |
| School Type | Single item |
| Engagement | EPOCH measure (Kern et al., 2016) |
| Perseverance | EPOCH measure (Kern et al., 2016) |
| Optimism | EPOCH measure (Kern et al., 2016) |
| Connectedness | EPOCH measure (Kern et al., 2016) |
| Happiness | EPOCH measure (Kern et al., 2016) |
| Grit | Grit scale (Duckworth et al., 2007) |
| Mastery-Approach | 2 × 2 achievement goal framework (Elliot et al., 2001) |
| Mastery-Avoidance | 2 × 2 achievement goal framework (Elliot et al., 2001) |
| Performance-Approach | 2 × 2 achievement goal framework (Elliot et al., 2001) |
| Performance-Avoidance | 2 × 2 achievement goal framework (Elliot et al., 2001) |
| School engagement | Healthy Pathways (Bevans et al., 2010) |
| Coping | single item |
| Empathy | single item |
| Self-Awareness | single item |
| Growth mindset | Growth mindset inventory (Dweck, 1996) |
| Anxiety | PROMIS pediatric short form (Varni et al., 2014) |
| Depression | PROMIS pediatric short form (Varni et al., 2014) |
| Health | PROMIS pediatric short form (Varni et al., 2014) |
| **8) Sample 8** | |
| Age | Single item |
| Gender | Single item |
| Grade | Single item |
| School | Single item |
| School Type | Single item |
| Engagement | EPOCH measure (Kern et al., 2016) |
| Perseverance | EPOCH measure (Kern et al., 2016) |
| Optimism | EPOCH measure (Kern et al., 2016) |
| Connectedness | EPOCH measure (Kern et al., 2016) |
| Happiness | EPOCH measure (Kern et al., 2016) |
| Anxiety | PROMIS pediatric short form (Varni et al., 2014) |
| Depression | PROMIS pediatric short form (Varni et al., 2014) |
| Health | PROMIS pediatric short form (Varni et al., 2014) |
| **9) Sample 9** | |
| Age | Single item |
| Gender | Single item |
| Grade | Single item |
| School | Single item |
| School Type | Single item |
| Engagement | EPOCH measure (Kern et al., 2016) |
| Perseverance | EPOCH measure (Kern et al., 2016) |
| Optimism | EPOCH measure (Kern et al., 2016) |
| Connectedness | EPOCH measure (Kern et al., 2016) |
| Happiness | EPOCH measure (Kern et al., 2016) |
| Grit | Grit scale (Duckworth et al., 2007) |
| Mastery-Approach | 2 × 2 achievement goal framework (Elliot et al., 2001) |
| Mastery-Avoidance | 2 × 2 achievement goal framework (Elliot et al., 2001) |
| Performance-Approach | 2 × 2 achievement goal framework (Elliot et al., 2001) |
| Performance-Avoidance | 2 × 2 achievement goal framework (Elliot et al., 2001) |
| School engagement | Healthy Pathways (Bevans et al., 2010) |
| School Performance | Healthy Pathways (Bevans et al., 2010) |
| Self-Efficacy | The GSES (Sherer & Adams, 1983) |
| Work Value | work value scale (Farrington, et al., 2012) |
| Resilience | 4 items from Brief Resilience Scale (Smith et al., 2008) |
| Relatedness to class | Relatedness Scale (Furrer & Skinner, 2003) |
| Growth mindset | Growth mindset inventory (Dweck, 1996) |
| Teacher Relation | Inclusion of others in the self scale (Aron et al., 1992) |
| Classroom Relation | Inclusion of others in the self scale (Aron et al., 1992) |
| Parent Relation | Inclusion of others in the self scale (Aron et al., 1992) |
| Anxiety | PROMIS pediatric short form (Varni et al., 2014) |
| Depression | PROMIS pediatric short form (Varni et al., 2014) |
| Health | PROMIS pediatric short form (Varni et al., 2014) |
| **10) Sample 10** | |
| Age | Single item |
| Gender | Single item |
| Grade | Single item |
| School | Single item |
| School Type | Single item |
| Engagement | EPOCH measure (Kern et al., 2016) |
| Perseverance | EPOCH measure (Kern et al., 2016) |
| Optimism | EPOCH measure (Kern et al., 2016) |
| Connectedness | EPOCH measure (Kern et al., 2016) |
| Happiness | EPOCH measure (Kern et al., 2016) |
| Anxiety | PROMIS pediatric short form (Varni et al., 2014) |
| Depression | PROMIS pediatric short form (Varni et al., 2014) |
| Health | PROMIS pediatric short form (Varni et al., 2014) |
| **11) Sample 11** | |
| Age | Single item |
| Gender | Single item |
| Grade | Single item |
| School | Single item |
| School Type | Single item |
| Engagement | EPOCH measure (Kern et al., 2016) |
| Perseverance | EPOCH measure (Kern et al., 2016) |
| Optimism | EPOCH measure (Kern et al., 2016) |
| Connectedness | EPOCH measure (Kern et al., 2016) |
| Happiness | EPOCH measure (Kern et al., 2016) |
| Growth mindset | Growth mindset inventory (Dweck, 1996) |
| School engagement | Healthy Pathways (Bevans et al., 2010) |
| Academic performance | Healthy Pathways (Bevans et al., 2010) |
| Resilience | Brief Resilience Scale (Smith et al., 2008) |
| Anxiety | PROMIS pediatric short form (Varni et al., 2014) |
| Depression | PROMIS pediatric short form (Varni et al., 2014) |
| Health | PROMIS pediatric short form (Varni et al., 2014) |

**Appendix 2**

*Comparing configural, weak, strong, and strict invariance across gender, school type, location, and area.*

|  | **Df** | **AIC** | **BIC** | **χ^2^** | **Δχ^2^** | **p** | **CFI** | **RMSEA** | **ΔCFI** | **ΔRMSEA** |
| --- | --- | --- | --- | --- | --- | --- | --- | --- | --- | --- |
| **Gender (male/ female)** | | | | | | | | | | |
| Configural model | 320 | 899841 | 900932 | 13752 |  |  | 0.928 | 0.068 | -- | -- |
| Weak invariance | 335 | 899832 | 900806 | 13772 | 22.398 | 0.098 | 0.928 | 0.067 | 0.000 | 0.002 |
| Strong invariance | 350 | 899985 | 900841 | 13955 | 188.933 | <.0001 | 0.927 | 0.066 | 0.001 | 0.001 |
| Strict invariance | 370 | 900156 | 900858 | 14167 | 138.807 | <.0001 | 0.926 | 0.064 | 0.001 | 0.001 |
| **School Type (primary only/ primary & secondary/ vocational & technical)** | | | | | | | | | | |
| Configural model | 480 | 895208 | 896844 | 13769 |  |  | 0.927 | 0.068 | -- | -- |
| Weak invariance | 510 | 895371 | 896774 | 13992 | 256.54 | <.0001 | 0.926 | 0.066 | 0.001 | 0.002 |
| Strong invariance | 540 | 896230 | 897398 | 14911 | 1216.56 | <.0001 | 0.921 | 0.066 | 0.005 | 0.000 |
| Strict invariance | 580 | 898760 | 899617 | 17521 | 2303.18 | <.0001 | 0.907 | 0.070 | 0.014 | 0.003 |
| **Location (Sinchuan Province, Tianing City, Hunan Province, Shanxi Province)** | | | | | | | | | | |
| Configural model | 640 | 895678 | 897859 | 14417 |  |  | 0.927 | 0.069 | -- | -- |
| Weak invariance | 685 | 895798 | 897628 | 14627 | 221.91 | <.0001 | 0.926 | 0.067 | 0.001 | 0.002 |
| Strong invariance | 730 | 896625 | 898105 | 15544 | 1098.56 | <.0001 | 0.921 | 0.067 | 0.005 | 0.000 |
| Strict invariance | 790 | 899425 | 900438 | 18465 | 2215.47 | <.0001 | 0.905 | 0.070 | 0.015 | 0.003 |
| **Area (urban/ rural)** | | | | | | | | | | |
| Configural model | 320 | 898387 | 899478 | 13926 |  |  | 0.926 | 0.069 | -- | -- |
| Weak invariance | 335 | 898435 | 899408 | 14004 | 81.73 | <.0001 | 0.926 | 0.067 | 0.000 | 0.001 |
| Strong invariance | 350 | 898764 | 899620 | 14363 | 376.32 | <.0001 | 0.924 | 0.067 | 0.002 | 0.001 |
| Strict invariance | 370 | 899813 | 900514 | 15452 | 626.89 | <.0001 | 0.918 | 0.067 | 0.006 | 0.001 |

*Note. Df = degrees of freedom, AIC = Akaike information criteria, BIC = Bayesian information criteria, CFI = confirmatory fit index, RMSEA = root mean square error of approximation. We determined invariance based on ΔCFI < .01.*

**Appendix 3**

*Mean comparisons across gender, school type, location, and area.*

| **Variable** | **Group Means (SD)** | | | | **t/F** | **Df** | **p** |
| --- | --- | --- | --- | --- | --- | --- | --- |
| **Gender** | **Males** | **Female** |  |  |  |  |  |
| Engagement | 3.68 (.91) | 3.64 (.87) |  |  | 3.21 | 17852 | 0.001 |
| Perseverance | 3.78 (.86) | 3.77 (.84) |  |  | 0.61 | 17852 | 0.54 |
| Optimism | 3.96 (.88) | 3.98 (.85) |  |  | -1.44 | 17852 | 0.15 |
| Connectedness | 4.00 (.86) | 4.12 (.80) |  |  | -9.71 | 17852 | 0 |
| Happiness | 4.04 (.92) | 4.06 (.89) |  |  | -1.55 | 17852 | 0.12 |
| Overall wellbeing | 3.89 (.75) | 3.91 (.70) |  |  | -2.02 | 17852 | 0.04 |
| **School Type** | **Primary**  **Only** | **Primary &**  **Secondary** | **Vocational/**  **Technical** |  |  |  |  |
| Engagement | 3.33 (.94) | 3.76 (.90) | 3.50 (.73) |  | 272.09 | 2, 17851 | <.001 |
| Perseverance | 3.42 (.92) | 3.87 (.86) | 3.61 (.70) |  | 316.88 | 2, 17851 | <.001 |
| Optimism | 3.76 (.90) | 4.07 (.87) | 3.71 (.77) |  | 307.72 | 2, 17851 | <.001 |
| Connectedness | 3.83 (.90) | 4.14 (.83) | 3.85 (.74) |  | 247.14 | 2, 17851 | <.001 |
| Happiness | 3.93 (.92) | 4.13 (.92) | 3.81 (.80) |  | 186.33 | 2, 17851 | <.001 |
| Overall wellbeing | 3.65 (.76) | 4.00 (.73) | 3.70 (.61) |  | 362.26 | 2, 17851 | <.001 |
| **Location** | **Sinchuan** | **Tianing City** | **Hunan** | **Shanxi** |  |  |  |
| Engagement | 3.76 (.90) | 3.66 (.97) | 3.53 (.88) | 3.60 (.75) | 77.13 | 3, 17850 | <.001 |
| Perseverance | 3.88 (.86) | 3.91 (.89) | 3.59 (.83) | 3.70 (.73) | 160.04 | 3, 17850 | <.001 |
| Optimism | 4.09 (.87) | 4.04 (.92) | 3.83 (.85) | 3.83 (.80) | 125.22 | 3, 17850 | <.001 |
| Connectedness | 4.13 (.83) | 4.08 (.89) | 3.96 (.83) | 3.95 (.75) | 62.01 | 3, 17850 | <.001 |
| Happiness | 4.14 (.93) | 4.15 (.95) | 3.91 (.88) | 3.95 (.80) | 85.50 | 3, 17850 | <.001 |
| Overall wellbeing | 4.00 (.73) | 3.97 (.77) | 3.76 (.71) | 3.81 (.63) | 137.91 | 3, 17850 | <.001 |
| **Area** | **Urban** | **Rural** |  |  |  |  |  |
| Engagement | 3.78 (.90) | 3.54 (.86) |  |  | -18.266 | 17852 | <.001 |
| Perseverance | 3.91 (.86) | 3.64 (.82) |  |  | -21.862 | 17852 | <.001 |
| Optimism | 4.10 (.86) | 3.84 (.85) |  |  | -20.085 | 17852 | <.001 |
| Connectedness | 4.15 (.84) | 3.96 (.82) |  |  | -15.101 | 17852 | <.001 |
| Happiness | 4.16 (.92) | 3.94 (.88) |  |  | -16.493 | 17852 | <.001 |
| Overall wellbeing | 4.02 (.73) | 3.78 (.71) |  |  | -22.023 | 17852 | <.001 |

**Appendix References**

Aron, A., Aron, E. N., & Smollan, D. (1992). Inclusion of other in the self scale and the structure of interpersonal closeness. Journal of personality and social psychology, 63(4), 596.

Bevans, K. B., Riley, A. W., & Forrest, C. B. (2010). Development of the healthy pathways child report scales. *Quality of Life Research, 19*, 1195–1214. <http://dx.doi.org/10.1007/s11136-010-9687-4>

Buhrmester, D., Furman, W., Wittenberg, M. T., & Reis, H. T. (1988). Five domains of interpersonal competence in peer relationships. *Journal of Personality and Social Psychology, 55*(6), 991-1008.

Cheung, G. W., & Rensvold, R. B. (2002). Evaluating goodness-of-fit indexes for testing measurement invariance. *Structural Equation Modeling, 9*, 233–255.

Deci, E. L., Vallerand, R. J., Pelletier, L. G., & Ryan, R. M. (1991). Motivation and education: The self-determination perspective. Educational psychologist, 26(3-4), 325-346.

Dweck, C. (2006). *Mindset: The new psychology of success*. New York: Random House.

Elliot, A. J., & McGregor, H. A. (2001). A 2 × 2 achievement goal framework. *Journal of Personality and Social Psychology, 80*(3), 501-519.

Farrington, C. A., Roderick, M., Allensworth, E., Nagaoka, J., Keyes, T. S., Johnson, D. W., & Beechum, N. O. (2012). *Teaching Adolescents to Become Learners: The Role of Noncognitive Factors in Shaping School Performance--A Critical Literature Review.* Consortium on Chicago School Research. 1313 East 60th Street, Chicago, IL 60637.

Furrer, C., & Skinner, E. (2003). Sense of relatedness as a factor in children's academic engagement and performance. *Journal of educational psychology*, *95*(1), 148.

Jorgensen, T. D., Pornprasertmanit, S., Miller, P., Schoemann, A., & Rosseel, Y. (2016). *Useful tools for structural equation modeleing: Package ‘semTools’* (version 0.4-14).

Ryan, R. M., & Connell, J. P. (1989). Perceived locus of causality and internalization: Examining reasons for acting in two domains. *Journal of personality and social psychology*, *57*(5), 749.

Schwarzer, R., & Jerusalem, M. (1995). Generalized Self-Efficacy scale. In J. Weinman, S. Wright, & M. Johnston, Measures in health psychology: A user’s portfolio. Causal and control beliefs (pp. 35-37). Windsor, UK: NFER-NELSON.

Smith, B. W., Dalen, J., Wiggins, K., Tooley, E., Christopher, P., & Berard, J. (2008). The brief resilience scale: Assessing the ability to bounce back. *International Journal of Behavioral Medicine, 15,* 194–200. <http://dx.doi.org/10.1080/10705500802222972>

Varni, J. W., Magnus, B., Stucky, B.D., Liu, Y., Quinn, H., Thissen, D.,…, & DeWalt, D. A. (2014). Psychometric properties of the PROMIS® pediatric scales: Precision, stability, and comparison of different scoring and administration options. *Quality of Life Research, 23*, 1233–1243. <http://dx.doi.org/10.1007/s11136-013-0544-0>
